# Therapeutic modulation of empathy: pharmacological, neurostimulation, and behavioral approaches

**DOI:** 10.3389/fpsyg.2026.1762816

**Published:** 2026-03-04

**Authors:** Sarfaraz K. Niazi

**Affiliations:** University of Illinois, Chicago, IL, United States

**Keywords:** empathy, ethics, mindfulness, neuromodulation, neurostimulation, oxytocin, therapeutic intervention, virtual reality

## Abstract

Empathy is a core neurobiological capacity that enables humans to perceive, understand, and respond to others’ experiences, yet its deliberate modulation as a therapeutic target is still in its early stages of scientific development. This manuscript presents a hybrid narrative–scoping review synthesizing evidence across pharmacological, neurostimulation, and behavioral approaches aimed at enhancing or restoring empathic functioning. A structured literature search covering 2014–2025 was conducted across PubMed, PsycINFO, Web of Science, and Scopus, with emphasis on post-2020 research. Evidence was organized using an author-defined tiered synthesis framework, intended to support integrative interpretation rather than formal evidence grading. Across modalities, empathy can be modulated, but observed effect sizes are typically small to moderate, heterogeneous, and frequently transient. Pharmacological approaches such as intranasal oxytocin demonstrate modest, context-dependent effects (*d* = 0.24, 95% CI: 0.02–0.46), while MDMA-assisted therapy yields larger but highly context-restricted benefits (*d* ≈ 0.91) in trauma-focused psychotherapy and faces unresolved regulatory barriers. Neurostimulation techniques, including transcranial magnetic stimulation (*d* ≈ 0.18–0.20), provide causal insights into empathy-related circuits but show limited durability of behavioral effects. Behavioral interventions—particularly mindfulness-based programs (*d* = 0.37, 95% CI: 0.16–0.58 for programs >24 h) and compassion-focused programs—exhibit the greatest scalability and sustainability. Overall, empathy represents a scientifically promising yet clinically constrained therapeutic target. Reliable translation will require larger trials, standardized and ecologically valid outcome measures, and careful integration of neurobiological and psychosocial frameworks.

## 1 Introduction

Empathy—the capacity to perceive, understand, and respond to the emotions and perspectives of others—is fundamental to human social functioning and underpins cooperation, caregiving, moral reasoning, and social cohesion (Decety et al., 2016; Singer and Klimecki, 2014). Over the past two decades, advances in affective neuroscience, neuroimaging, and social cognition have clarified the neural circuits, neurochemical systems, and developmental processes that support empathic behavior (Kogler et al., 2020; Tian et al., 2025). Recent integrative syntheses further demonstrate that empathy-related neural systems can be modulated by pharmacological, neurostimulation, and behavioral interventions, although translational effect sizes remain modest and context dependent (Abellaneda-Pérez et al., 2024; Brooker et al., 2024). These advances have stimulated increasing interest in whether empathy can be deliberately modulated for therapeutic, educational, or societal benefit.

Despite this growing interest, translational progress has been limited. Many interventions demonstrate statistically significant effects under laboratory conditions but yield small effect sizes, short-lived changes, or limited generalization to real-world social behavior (Holleman et al., 2020; Lu and Lau, 2025). Early optimism surrounding pharmacological modulation—particularly intranasal oxytocin—has been tempered by replication failures, strong contextual moderation, and population-specific effects (Bakermans-Kranenburg and van IJzendoorn, 2013; Stark et al., 2023). Similar constraints affect neurostimulation and technologically mediated behavioral approaches. These limitations underscore the need for integrative evaluation of empathy-modulation strategies and realistic expectations regarding clinical applicability.

### 1.1 Empathy as a multicomponent construct

Empathy is not a unitary phenomenon but comprises interacting yet dissociable components that rely on partially distinct neural substrates (Singer and Klimecki, 2014; Kogler et al., 2020). Affective empathy refers to the automatic sharing or resonance with another individual’s emotional state and is supported by limbic and paralimbic structures, including the anterior insula, anterior cingulate cortex, amygdala, and somatosensory cortices (Allen et al., 2017; Paradiso et al., 2021). These regions integrate interoceptive signals with externally perceived emotional cues, generating subjective feeling states that support empathic concern.

In contrast, cognitive empathy, often termed perspective-taking or mentalizing, involves inferential processes that enable individuals to represent and reason about others’ mental states. Cognitive empathy depends on prefrontal–temporal networks, particularly the right temporoparietal junction (rTPJ), the medial prefrontal cortex, the superior temporal sulcus, and the temporal poles (Chou and Chen, 2021; Jiang et al., 2022). While affective and cognitive empathy interact dynamically in real-world social behavior, they can be selectively impaired or enhanced and are differentially sensitive to intervention modalities.

This neurobiological distinction has major implications for the development of interventions. Pharmacological and neurostimulation approaches show differential effects on affective versus cognitive empathy components, and behavioral interventions often target motivational, regulatory, and cognitive processes differentially. Understanding these separations is crucial for designing selective, mechanism-informed therapeutic strategies that match intervention modality to the specific empathic deficit requiring remediation.

### 1.2 Clinical and societal significance

Alterations in empathic functioning contribute to functional impairment across a broad range of psychiatric and neurodevelopmental conditions. Autism spectrum conditions are often associated with difficulties in cognitive empathy and social reciprocity, whereas psychopathy is characterized by relatively preserved cognitive empathy alongside reduced affective empathy (Rinaldi et al., 2021). Borderline personality disorder presents with empathy dysregulation, and schizophrenia often involves mentalizing impairments alongside negative symptoms. These clinical associations have motivated interest in targeted empathy-enhancing interventions (Moudatsou et al., 2020).

Beyond psychiatric and neurodevelopmental disorders, empathy plays a central role in healthcare delivery, where higher clinician empathy is consistently associated with improved therapeutic alliance, increased patient satisfaction, better adherence, and improved outcomes (Nembhard et al., 2023; Araújo et al., 2025). Empathy-focused educational and behavioral interventions have therefore been increasingly adopted in medical and allied health training programs, with systematic reviews demonstrating moderate but reliable improvements in empathic skills (Araújo et al., 2025; Martins et al., 2025). Educational systems similarly acknowledge empathy as a fundamental social–emotional competency associated with mental health, academic engagement, and prosocial behavior (Cipriano et al., 2023). These findings have stimulated interest in scalable interventions that augment empathic capacity within both professional and general populations.

### 1.3 Methodological challenges

Despite extensive research activity, the science of empathy-modulation encounters ongoing methodological challenges. Most investigations depend on self-report questionnaires or brief laboratory tasks that inadequately capture the dynamic, context-dependent nature of social interactions in real-world settings (Lima and Osório, 2021). The heterogeneity of measurement approaches hampers comparability across studies and may exaggerate apparent effect sizes. Furthermore, individual differences—including genetic variation, sex, age, cultural background, and baseline empathy—contribute significant variability in responses to interventions (Chander et al., 2022; Ferguson et al., 2024).

Moreover, improvements observed in controlled experimental settings often fail to translate into sustained prosocial behavior in naturalistic environments, raising concerns about ecological validity and long-term clinical utility (Holleman et al., 2020; Winter et al., 2020). These limitations necessitate cautious interpretation of reported effects and emphasize the need for more robust translational frameworks.

### 1.4 Scope and objectives

This review synthesizes evidence from pharmacological, neurostimulation, and behavioral approaches pertaining to the modulation of empathy. Rather than providing prescriptive clinical guidelines, the objectives are to: (i) compile current evidence through a transparent, integrative framework; (ii) assess the magnitude, durability, and generalizability of reported effects; (iii) elucidate translational limitations and ethical considerations; and (iv) delineate priorities for future research. By contextualizing empathy modulation within its neurobiological, psychological, and sociocultural dimensions, this review endeavors to offer a balanced evaluation of current knowledge, existing uncertainties, and the prerequisites for responsible clinical application.

## 2 Review design and methods

### 2.1 Review type clarification

The present synthesis incorporates evidence from large-scale randomized trials, mechanistic neuroimaging studies, and translational safety guidelines to contextualize emerging empathy-modulation technologies (Martin et al., 2024; Murphy et al., 2025). This manuscript is a hybrid narrative–scoping review. While a structured literature search and explicit inclusion criteria are presented to enhance transparency and breadth, the primary goal is conceptual integration and translational synthesis, not exhaustive systematic review or formal evidence grading. Accordingly, established grading systems such as PRISMA or GRADE were not applied, as the intent is not to develop guidelines but to conduct critical synthesis across heterogeneous intervention modalities.

### 2.2 Literature search strategy

A comprehensive search was systematically conducted within PubMed, PsycINFO, Web of Science, and Scopus databases for scholarly articles published between January 2014 and March 2025. Search strategies incorporated combinations of keywords such as empathy, empathic, modulation, intervention, oxytocin, MDMA, neurostimulation, transcranial magnetic stimulation, transcranial direct current stimulation, mindfulness, compassion, and virtual reality. The reference lists of major meta-analyses, systematic reviews, and high-impact trials were meticulously screened to identify additional pertinent publications. Emphasis was placed on large-scale randomized controlled trials, registered meta-analyses and systematic reviews, studies utilizing neuroimaging techniques or established neural biomarkers, and research with explicit operational definitions of empathy constructs.

### 2.3 Inclusion and exclusion criteria

Included studies were peer-reviewed human investigations or systematic reviews that explicitly measured affective empathy, cognitive empathy, or closely related constructs such as compassion or empathic concern. Animal studies were consulted solely to contextualize neurobiological mechanisms and were not used to infer clinical effect sizes. Single-case reports, non-peer-reviewed sources, conference abstracts, and studies lacking empathy-specific outcomes were excluded.

### 2.4 Tiered evidence synthesis framework

To facilitate integrative interpretation across diverse literatures, evidence was organized using an author-defined three-tier synthesis framework, applied solely as an interpretive heuristic:

Tier 1: Meta-analyses or randomized controlled trials with ≥500 participants.

Tier 2: Moderate-sized trials (100–499 participants) and mechanistic human studies.

Tier 3: Emerging, exploratory, or proof-of-concept research (<100 participants).

The ≥500-participant threshold for Tier 1 was selected to reduce small-study bias, enhance stability of effect-size estimates, and support cautious translational inference. This framework does not constitute formal evidence grading and is used only for synthesis and contextual guidance, not for prescriptive recommendations (Table 1).

**Table 1:** Tiered evidence synthesis framework (author-defined).

Tier	Criteria	Typical study types	Purpose in this review	Representative references
Tier 1	≥500 participants in aggregate	Meta-analyses; large randomized controlled trials	Support cautious translational interpretation and comparative evaluation across modalities	Hu et al. (2022), Stark et al. (2023), and Cipriano et al. (2023)
Tier 2	100–499 participants	Moderate-sized RCTs; mechanistic human studies	Elucidate mechanisms and boundary conditions of effects	Bahji et al. (2021) and Paulus and Meinken (2022)
Tier 3	<100 participants or exploratory designs	Pilot studies; proof-of-concept trials; emerging technologies	Hypothesis generation and identification of future research directions	Legon et al. (2024) and Violante et al. (2023)

This tiered framework is an author-defined synthesis heuristic, not a formal evidence-grading system (e.g., GRADE or PRISMA). The ≥500-participant threshold for Tier 1 was selected to reduce small-study bias and enhance stability of effect-size estimates. Tiers are used solely for integrative synthesis and translational context, not for prescriptive clinical recommendations.

## 3 Neural architecture and mechanistic foundations of empathy

Empathy arises from distributed neural systems that integrate affective resonance, interoception, perspective-taking, motivational salience, and social prediction. Converging evidence from neuroimaging, lesion studies, and neuromodulation demonstrates that empathic processes are supported by partially dissociable yet dynamically interacting circuits, providing a mechanistic basis for selective modulation by pharmacological, neurostimulation, and behavioral interventions (Singer and Klimecki, 2014; Kogler et al., 2020; Paradiso et al., 2021; Brooker et al., 2024). Animal and human mechanistic studies demonstrate that empathic processing is supported by conserved neural circuits across species, providing a biological rationale for translational intervention strategies (Brooker et al., 2024; Cox et al., 2022; Kuroda et al., 2024). Developmental neuroimaging studies further indicate that empathy-related circuitry undergoes prolonged maturation and remains plastic across the lifespan, with dopaminergic and mesolimbic pathways playing a modulatory role (Liang et al., 2025).

### 3.1 Affective empathy: limbic and paralimbic circuits

Affective empathy involves the rapid and largely automatic sharing of another individual’s emotional state. Functional neuroimaging consistently implicates the anterior insula and anterior cingulate cortex as core hubs supporting affective resonance, emotional salience, and motivational readiness to respond to others’ distress (Singer et al., 2004; Allen et al., 2017; Rolls, 2019). The anterior insula integrates interoceptive signals with externally perceived emotional cues, generating subjective feeling states that underlie empathic concern (Craig, 2009; Allen et al., 2017). Recent neuroimaging evidence using intracranial recordings demonstrates that the insula uniquely exhibits factorized coding of emotion and agency, allowing cross-person generalization of emotional states—a computational property essential for empathic transfer (Haynes et al., 2025).

The anterior cingulate cortex contributes to the motivational and action-oriented aspects of empathy, linking emotional salience to behavioral responses through dense connectivity with limbic and reward-related structures (Rolls, 2019). The amygdala modulates threat detection and emotional learning, shaping empathic responses based on contextual relevance and prior experience (Marsh et al., 2021). Together, these regions form a limbic–paralimbic network that supports emotional contagion and empathic arousal.

Neurochemical systems modulate affective empathy within these circuits. Oxytocinergic signaling influences salience attribution and affiliative motivation by modulating amygdala–insula connectivity, while serotonergic systems regulate emotional responsiveness and affective tone (Shamay-Tsoory and Abu-Akel, 2016; Marsh et al., 2021). These mechanisms help explain why pharmacological interventions often produce context-dependent effects rather than uniform empathy enhancement.

### 3.2 Cognitive empathy: prefrontal–temporal networks

Cognitive empathy, often operationalized as perspective-taking or theory of mind, depends on higher-order inferential processes that allow individuals to represent others’ beliefs, intentions, and emotions. The right temporoparietal junction (rTPJ) is consistently identified as a central node in this network, supporting self–other distinction and mental state attribution (Saxe and Kanwisher, 2003; Chou and Chen, 2021). Disruption of rTPJ activity using transcranial magnetic stimulation selectively alters moral judgment and reduces sensitivity to others’ intentions, providing causal evidence for its role in cognitive empathy (Chou and Chen, 2021; Jiang et al., 2022).

The medial prefrontal cortex supports integration of contextual information, trait inference, and social evaluation, while the superior temporal sulcus contributes to the processing of biological motion and socially relevant cues (Kogler et al., 2020; Tian et al., 2025; Liang et al., 2025). These regions interact dynamically during complex social reasoning tasks, enabling flexible perspective-taking.

Unlike affective empathy, cognitive empathy is less strongly linked to immediate emotional arousal and more amenable to deliberate training and cognitive strategies. This distinction helps explain why neurostimulation and behavioral interventions more reliably influence cognitive than affective components of empathy.

### 3.3 Developmental, individual, and cultural modulators

Empathy-related neural systems are shaped by development, experience, and sociocultural context. Longitudinal studies indicate that empathic processing undergoes protracted maturation from adolescence into older adulthood, with age-related changes in prefrontal–temporal connectivity influencing cognitive empathy capacity (Ferguson et al., 2024; Kuroda et al., 2024). Sex differences in empathic responding have been reported, with females on average showing higher affective empathy and greater limbic responsivity, though effect sizes are modest and context-dependent (Christov-Moore et al., 2014).

**Figure 1:**
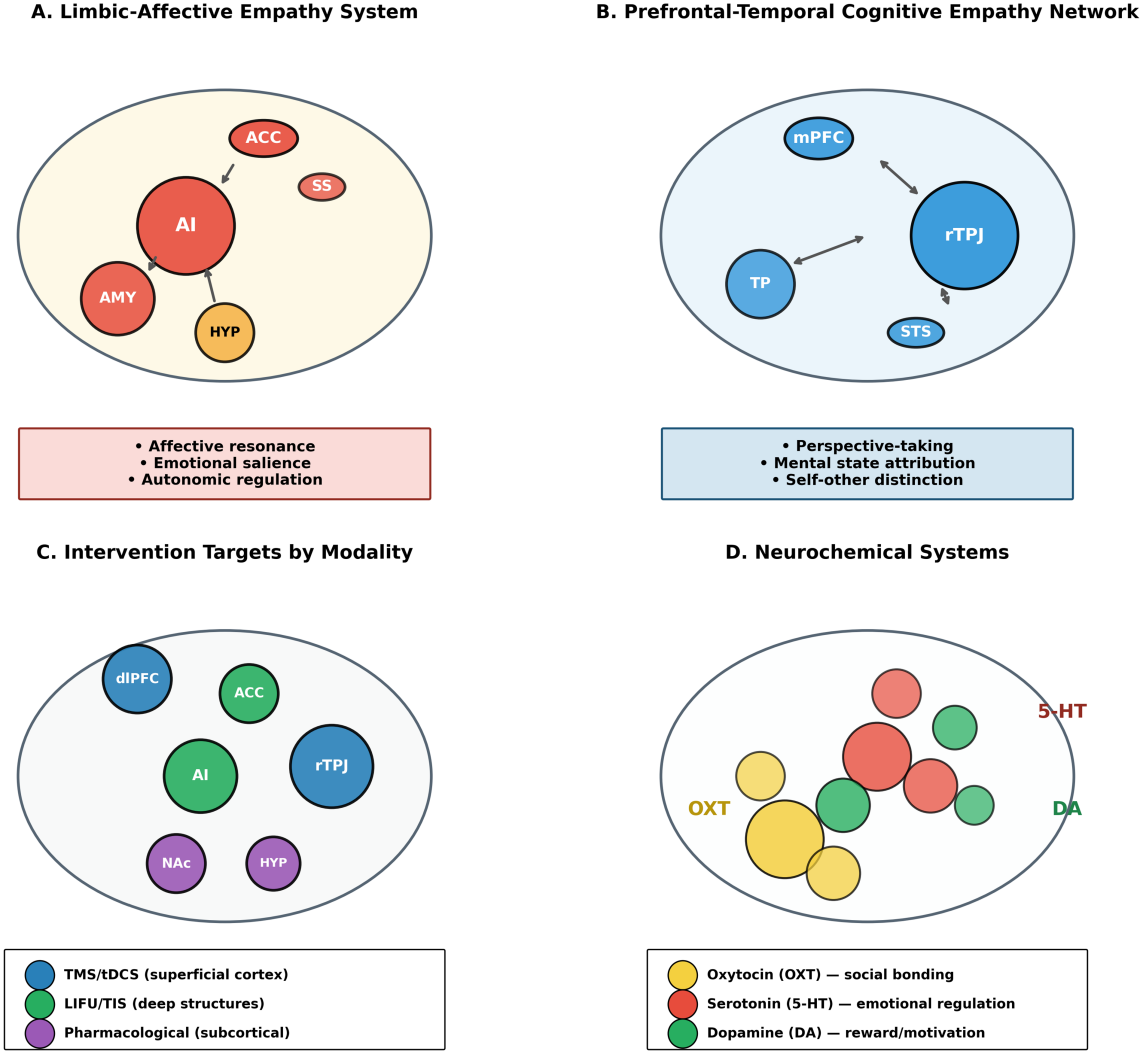
Neural architecture of empathy: integrated circuitry and intervention targets. This figure presents a conceptual synthesis derived from the empirical literature, not an anatomical atlas or primary neuroimaging result. **(A)** Depicts the limbic-affective empathy system, showing the anterior insula as the central hub that integrates interoceptive signals with emotional perception inputs, with bidirectional connectivity to the amygdala for salience processing and to hypothalamic nuclei for autonomic regulation. **(B)** Illustrates the prefrontal-temporal cognitive empathy network, centered on the right temporoparietal junction (rTPJ), with connections to the medial prefrontal cortex for self-other distinction and mental-state inference. **(C)** Presents a sagittal brain view with color-coded regions indicating primary intervention targets: blue regions denote optimal targets for transcranial magnetic stimulation and transcranial direct current stimulation (superficial cortical structures including rTPJ and dlPFC); green regions indicate emerging targets for low-intensity focused ultrasound and temporal interference stimulation (deeper structures including anterior insula and ACC); and purple regions highlight structures primarily modulated through pharmacological approaches. **(D)** Provides a neurochemical overlay showing the distribution of oxytocinergic, serotonergic, and dopaminergic receptor densities relevant to empathy modulation. The figure is intended to support integrative interpretation and does not imply precise anatomical boundaries or clinical efficacy.

Genetic variation further moderates empathic responsiveness. Polymorphisms in the oxytocin receptor gene (e.g., rs53576) have been associated with individual differences in empathy and social sensitivity, though effect sizes are small and replication across populations has been inconsistent (Bakermans-Kranenburg and van IJzendoorn, 2013; Chander et al., 2022). Cultural norms and socialization practices also shape empathic expression, influencing both baseline empathy and responsiveness to interventions (Eichbaum et al., 2023; Jami et al., 2024) (Figure 1 and Table 2).

**Table 2:** Neural targets for empathy modulation.

**Empathy component**	**Primary neural targets**	**Neurochemical systems**	**Interventions most likely to modulate**
Affective resonance	Anterior insula, ACC, amygdala	Oxytocin, serotonin (5-HT1A)	LIFU, intranasal oxytocin, mindfulness training
Cognitive perspective-taking	rTPJ, mPFC, STS	Dopamine (mesocortical)	tDCS/TMS over rTPJ, VR perspective-taking, compassion training
Prosocial motivation	Nucleus accumbens, VTA	Dopamine, serotonin (NAc)	MDMA-assisted therapy, reward-based behavioral paradigms
Affective regulation	vmPFC, dlPFC, ACC	Serotonin, GABA	Mindfulness, compassion-based therapy, neurofeedback
Empathic accuracy	rTPJ, AI, temporal pole	Oxytocin (context-dependent)	Social skills training, VR simulation, tDCS

ACC = anterior cingulate cortex; rTPJ = right temporoparietal junction; mPFC = medial prefrontal cortex; STS = superior temporal sulcus; VTA = ventral tegmental area; NAc = nucleus accumbens; vmPFC = ventromedial prefrontal cortex; dlPFC = dorsolateral prefrontal cortex; AI = anterior insula; LIFU = low-intensity focused ultrasound; tDCS = transcranial direct current stimulation; TMS = transcranial magnetic stimulation; VR = virtual reality.

## 4 Pharmacological modulation of empathy

Pharmacological approaches to empathy modulation target neurochemical systems involved in social salience, emotional resonance, and motivational processing. While these interventions provide important mechanistic insights, translational outcomes have been constrained by modest effect sizes, strong contextual dependence, and limited durability. This section evaluates the most extensively studied pharmacological agents using the tiered synthesis framework described above. Recent systematic and mechanistic reviews emphasize that pharmacological modulation of empathy produces small-to-moderate effects that are highly context dependent, with limited durability outside structured therapeutic settings (Modak et al., 2024; Saccenti et al., 2024).

### 4.1 Oxytocin: small effects with substantial moderation

Intranasal oxytocin has been the most intensively investigated pharmacological candidate for empathy enhancement, motivated by its established role in social bonding, attachment, and affiliative behavior (Shamay-Tsoory and Abu-Akel, 2016; Matsushita and Nishiki, 2025; Yuan et al., 2025). Early small-sample studies reported increases in emotion recognition, trust, and empathic concern, generating substantial enthusiasm for oxytocin as a potential “pro-social” agent.

However, subsequent large-scale meta-analyses have substantially tempered these expectations. A comprehensive meta-analytic review encompassing more than 1,100 participants found that intranasal oxytocin produces small effects on empathy-related outcomes, with a pooled effect size of approximately *d* = 0.24 (95% CI: 0.02–0.46), and wide confidence intervals indicating substantial heterogeneity (Stark et al., 2023). Importantly, publication-bias–corrected estimates were markedly smaller than those reported earlier, suggesting inflated effect sizes in underpowered studies.

Oxytocin effects are highly context-dependent. Experimental work demonstrates that oxytocin can enhance empathy and cooperation toward in-group members while simultaneously increasing defensive or exclusionary responses toward perceived out-groups (Marsh et al., 2021). These findings align with the social salience hypothesis, which posits that oxytocin amplifies the perceived relevance of social cues rather than uniformly increasing prosociality (Shamay-Tsoory and Abu-Akel, 2016). Genetic moderators further complicate interpretation: polymorphisms in the oxytocin receptor gene (e.g., rs53576) have been associated with differential responsiveness, though effect sizes are small and replication across ethnic and cultural groups has been inconsistent (Bakermans-Kranenburg and van IJzendoorn, 2013; Chander et al., 2022).

Taken together, Tier 1 evidence indicates that oxytocin does not reliably produce durable or generalized empathy enhancement and is unlikely to serve as a standalone therapeutic intervention.

### 4.2 MDMA-assisted therapy: large but context-restricted effects

MDMA (3,4-methylenedioxymethamphetamine) produces robust acute empathogenic effects characterized by increased emotional openness, affiliative motivation, and perceived social connectedness (Rein et al., 2024). Unlike oxytocin, MDMA exerts its effects primarily through potent serotonin release, with secondary effects on dopamine and norepinephrine signaling, and indirect activation of oxytocinergic pathways (Vaslavski et al., 2025). A landmark 2024 study demonstrated that MDMA enhances empathy-like behaviors specifically via serotonin release in the nucleus accumbens, with direct nucleus accumbens infusion alone sufficient to reproduce systemic empathogenic effects (Rein et al., 2024).

Tier 1 evidence from large randomized controlled trials demonstrates that MDMA-assisted psychotherapy yields large effect sizes for symptom reduction in post-traumatic stress disorder, with pooled estimates approaching d ≈ 0.91 (Mitchell et al., 2021, 2023). Phase 3 clinical trials demonstrated significant reductions in PTSD symptoms, with 67–71% of participants no longer meeting diagnostic criteria compared to 32–47% in placebo groups. These effects are widely interpreted as reflecting facilitation of therapeutic engagement, emotional processing, and trust within a structured psychotherapeutic context.

Despite these promising results, MDMA’s translational scope is highly constrained. Benefits appear tightly coupled to the therapeutic setting and do not generalize to unsupervised contexts. In August 2024, the U.S. Food and Drug Administration declined approval of MDMA-assisted therapy, citing concerns related to functional unblinding, therapist expectancy effects, durability of benefit, and safety oversight (Reardon, 2024; Wolfgang et al., 2025). These concerns underscore that MDMA’s empathogenic effects, while robust, do not equate to generalized or sustained empathy enhancement and cannot currently be extrapolated to broader clinical or enhancement applications.

### 4.3 Psilocybin and other serotonergic psychedelics

Classic serotonergic psychedelics, particularly psilocybin, have emerged as candidates for modulating emotional and social processing. Psilocybin primarily acts as a 5-HT2A receptor agonist, producing alterations in affective processing, self-referential cognition, and social connectedness (Jungwirth et al., 2025).

A randomized, placebo-controlled trial in patients with major depressive disorder demonstrated that psilocybin administration led to significant increases in emotional empathy, particularly toward positively valenced stimuli, with effects persisting for several weeks post-treatment (Jungwirth et al., 2025). Notably, cognitive empathy was largely unaffected, suggesting selective modulation of affective components. While these findings are promising, sample sizes remain modest, and empathy outcomes are secondary endpoints. Accordingly, psilocybin currently qualifies as Tier 2 evidence for empathy modulation.

### 4.4 Serotonergic antidepressants: paradoxical effects

In contrast to psychedelic-assisted interventions, conventional serotonergic antidepressants illustrate the complexity of neurochemical modulation of empathy. Contrary to assumptions that depression impairs empathy, longitudinal neuroimaging studies demonstrate that antidepressant treatment—rather than depression itself—leads to reductions in behavioral and neural responses to pain empathy. After 3 months of antidepressant therapy, patients showed significant decreases in both affective empathy and activity in brain regions associated with empathy for pain (Rütgen et al., 2019).

Clinical evidence indicates that 40–60% of individuals taking SSRI antidepressants experience “SSRI-induced indifference” or “emotional blunting,” characterized by feeling emotionally detached with reduced sympathy and empathy (Ma et al., 2021; Christensen et al., 2022). This paradoxical effect suggests that while SSRIs effectively treat depression, they may simultaneously reduce empathic responding through mechanisms affecting reinforcement learning and reward sensitivity (Langley et al., 2023). SSRIs, therefore, cannot be considered empathy-enhancing; instead, they illustrate the fragility of empathy circuits to serotonergic modulation and the importance of understanding neurochemical specificity in intervention development (Table 3).

**Table 3:** Pharmacological interventions and reported effects on empathy.

Intervention	Primary mechanism	Empathy component affected	Reported effect size	Evidence tier	Key references
Intranasal oxytocin	Oxytocin receptor modulation; social salience	Affective empathy (context-dependent)	*d* = 0.24 (95% CI: 0.02–0.46)	Tier 1	Bakermans-Kranenburg and van IJzendoorn (2013) and Stark et al., 2023
MDMA-assisted therapy	Serotonin release; social reward enhancement	Affective empathy; affiliative motivation	*d* ≈ 0.91 (large, context-restricted)	Tier 1	Mitchell et al. (2021, 2023) and Rein et al. (2024)
Psilocybin	5-HT2A agonism; affective processing	Emotional empathy (selective)	Moderate (secondary outcomes)	Tier 2	Jungwirth et al. (2025)
SSRIs	Serotonin reuptake inhibition	Affective empathy (often reduced)	Negative or blunting effects	Tier 1–2	Rütgen et al. (2019), Ma et al. (2021), and Christensen et al. (2022)

Effect sizes reflect pooled or representative estimates from meta-analyses or large trials where available. The tier designation follows the author-defined synthesis framework and is intended solely for integrative interpretation, not for prescriptive guidance. SSRI = selective serotonin reuptake inhibitor.

## 5 Neurostimulation approaches to empathy modulation

Neurostimulation techniques enable causal manipulation of neural circuits implicated in empathic processing. Compared with pharmacological interventions, neurostimulation provides greater spatial specificity but is constrained by modest effect sizes, substantial interindividual variability, and limited durability of behavioral effects. This section evaluates established noninvasive techniques alongside emerging deep neuromodulation approaches. Consensus safety and efficacy reviews indicate that noninvasive neurostimulation methods provide valuable causal insights into empathy-related circuits but are not yet suitable as standalone clinical interventions (Martin et al., 2024; Murphy et al., 2025).

### 5.1 Transcranial magnetic stimulation (TMS)

Transcranial magnetic stimulation modulates cortical excitability through focal magnetic pulses and has been widely used to investigate the causal role of empathy-related brain regions. Meta-analytic evidence from 22 studies indicates that repetitive TMS applied to empathy-relevant cortical targets produces small but statistically significant effects on empathy-related outcomes, with pooled effect sizes around d ≈ 0.18–0.20 (Yang et al., 2018). Effects are most consistently observed for cognitive empathy, particularly when stimulating the right temporoparietal junction (rTPJ).

Experimental disruption of rTPJ activity using TMS alters moral judgment and reduces sensitivity to others’ intentions, providing causal evidence for its role in perspective-taking and mental state attribution (Chou and Chen, 2021; Jiang et al., 2022). Recent neurophysiological studies confirm that low-frequency (1 Hz) TMS over the rTPJ during empathy induction increases withdrawal of parasympathetic nervous system activity, providing enhanced evidence for the temporoparietal junction’s causal role in empathic responding (Miller et al., 2020).

Despite these insights, the translational utility of TMS remains limited. Effects typically persist for minutes to an hour post-stimulation, and repeated-session protocols have yielded inconsistent behavioral outcomes. The 2021 International Federation of Clinical Neurophysiology safety guidelines confirm the TMS safety profile with extremely rare seizure incidence (<0.1% in clinical populations), providing enhanced confidence for clinical applications (Rossi et al., 2021). However, TMS’s inability to reach deep limbic structures limits its impact on affective empathy. Given these time constraints, TMS for empathy enhancement currently serves specialized research and clinical functions rather than as a standalone therapeutic intervention.

### 5.2 Transcranial direct current stimulation (tDCS)

Transcranial direct current stimulation applies weak electrical currents to modulate cortical excitability. Compared with TMS, tDCS is less spatially precise but more portable and scalable. A systematic review and meta-analysis of randomized controlled trials reported small, statistically significant effects of tDCS on empathy-related measures, with anodal stimulation over the rTPJ preferentially enhancing cognitive empathy in laboratory tasks (Bahji et al., 2021).

Contemporary research confirms that anodal tDCS over the right temporoparietal junction enhances prosocial learning by affecting cognitive empathy processes, with participants receiving active stimulation (*n* = 75) showing significantly improved learning performance in prosocial-learning conditions compared to self-learning contexts (Zhang et al., 2024). Clinical applications show preliminary benefits in autism spectrum disorder, where tDCS significantly improves Empathy Quotient scores and facial emotion recognition for threat-related emotions (Wilson et al., 2021). Research also investigates tDCS as an intervention to improve empathic abilities and reduce violent behavior in forensic offenders, particularly when targeting the ventromedial prefrontal cortex (Sergiou et al., 2020).

As with TMS, tDCS effects are generally transient and highly variable across individuals. Polarity-specific and montage-specific effects are difficult to reproduce consistently, limiting clinical translation (Schwertfeger et al., 2023; Son et al., 2025; Saccenti et al., 2024).

### 5.3 Emerging deep neuromodulation techniques

Recent technological advances have enabled noninvasive targeting of deeper brain structures implicated in empathy, though evidence remains exploratory.

Low-intensity focused ultrasound (LIFU) has emerged as a transformative non-invasive neuromodulation technique, offering millimeter-sized focal volumes with adjustable focal lengths that can target deep human brain circuitry with unprecedented precision. Unlike other non-invasive brain stimulation techniques, focused ultrasound provides focal deep brain targeting, multi-target stimulation capabilities, and neuromodulatory effects lasting from milliseconds to hours after sonication (Pellow et al., 2024; Kim et al., 2024; Martin et al., 2024). Human studies demonstrate that LIFU can modulate activity in deep structures such as the thalamus and insula, affecting pain perception and autonomic responses (Badran et al., 2020; Legon et al., 2024; Murphy et al., 2025). The European Research Council-funded HelpUS project specifically pioneers focused ultrasound as a novel non-invasive deep brain stimulation method for causal investigation of empathy-related brain processes in moral learning and decision-making (Gazzola, 2018). While these findings suggest mechanistic relevance for affective empathy, no controlled human trials have yet demonstrated direct empathy enhancement using LIFU. Accordingly, current evidence is best classified as Tier 3.

**Figure 2:**
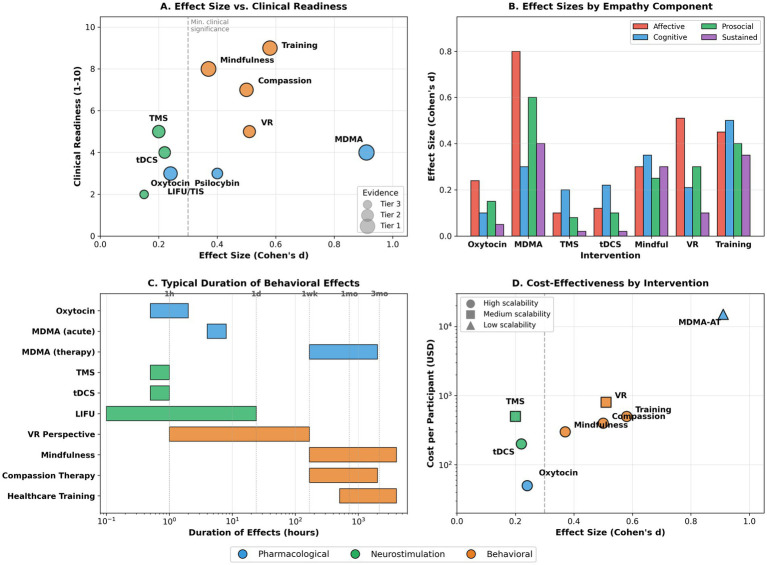
Comparative landscape of neurostimulation interventions for empathy. This figure presents a literature-derived comparative schematic of neurostimulation approaches to empathy modulation. **(A)** Displays a bubble chart plotting effect size (*x*-axis: Cohen’s *d*, 0–1.0) against clinical readiness level (*y*-axis: 1–10, from experimental to established clinical practice), with bubble size representing evidence quality and bubble color indicating intervention category. **(B)** Presents a stacked bar chart comparing effect sizes across empathy components (affective, cognitive, prosocial motivation, sustained behavioral change) for each intervention category. **(C)** Shows a timeline of effect duration (*x*-axis in hours/days/weeks/months) for each intervention type, with annotations indicating typical decay patterns. **(D)** Provides a cost-effectiveness analysis plotting the estimated cost per participant against the effect size. This figure is a conceptual synthesis derived from published meta-analyses, randomized trials, and mechanistic studies (Yang et al., 2018; Bahji et al., 2021; Rossi et al., 2021; Legon et al., 2024). It does not represent primary data or quantitative meta-analysis and is intended solely to facilitate integrative interpretation (Table 4).

Temporal interference stimulation (TIS) represents a breakthrough non-invasive technique for steerable deep brain stimulation using multiple kHz-range electric fields with different frequencies within neural activity ranges. Human validation studies demonstrate that TIS can modulate hippocampal activity and associated cognitive effects focally, while sparing the superficial cortex (Violante et al., 2023). Open-label pilot studies show TIS targeting the right nucleus accumbens alleviates negative symptoms and improves cognitive function in schizophrenia patients, with participants demonstrating significant improvements in Positive and Negative Syndrome Scale negative subscale scores over 90 days (Wang et al., 2025). Recent safety studies involving over 250 TIS sessions confirm excellent tolerability and safety profiles (Vassiliadis et al., 2024; Modak et al., 2024). As with LIFU, TIS remains exploratory regarding empathy modulation (Figure 2 and Table 4).

**Table 4:** Neurostimulation approaches and evidence for empathy modulation.

Technique	Primary targets	Empathy component	Effect size	Duration	Tier	Key references
TMS	rTPJ, mPFC	Cognitive empathy	*d* ≈ 0.18–0.20 (small)	Minutes–1 h	Tier 1	Yang et al. (2018) and Chou and Chen (2021)
tDCS	rTPJ	Cognitive empathy; prosocial learning	*d* ≈ 0.18–0.25 (small)	Minutes–1 h	Tier 1–2	Bahji et al. (2021) and Zhang et al. (2024)
LIFU	Insula, thalamus	Mechanistic relevance to affective empathy	Not established	Variable (ms to hours)	Tier 3	Badran et al. (2020) and Legon et al. (2024)
TIS	Hippocampus, mesolimbic regions	Social cognition (indirect)	Not established	Unknown	Tier 3	Violante et al. (2023) and Wang et al. (2025)

TMS = transcranial magnetic stimulation; tDCS = transcranial direct current stimulation; LIFU = low-intensity focused ultrasound; TIS = temporal interference stimulation; rTPJ = right temporoparietal junction; mPFC = medial prefrontal cortex.

## 6 Behavioral interventions for empathy enhancement

Behavioral interventions represent the most mature and scalable approaches to empathy modulation. Unlike pharmacological and neurostimulation strategies—which target specific neurochemical systems or neural circuits—behavioral approaches operate through experience-dependent plasticity, influencing attentional, emotional, motivational, and interpersonal processes over time. As a result, they demonstrate stronger ecological validity and fewer safety concerns, though effect sizes remain modest and dependent on sustained engagement. Behavioral and educational approaches remain the most scalable and ethically robust methods for empathy enhancement, particularly in professional and educational settings (Schutte and Stilinović, 2017; Shi and Cheung, 2024).

### 6.1 Mindfulness-based interventions

Mindfulness-based interventions, including mindfulness-based stress reduction, loving-kindness meditation, and compassion meditation, are among the most extensively studied behavioral approaches to empathy enhancement. These interventions emphasize attentional regulation, interoceptive awareness, and non-reactive emotional processing, mechanisms that align closely with neural systems supporting affective regulation and empathic concern (Singer and Klimecki, 2014; Calderone et al., 2024; Abellaneda-Pérez et al., 2024).

Tier 1 meta-analytic evidence indicates that mindfulness-based programs produce small-to-moderate improvements in empathy, with a pooled effect size of approximately *d* = 0.37 (95% CI: 0.16–0.58), and significantly stronger effects observed in interventions exceeding 24 total hours of training (Hu et al., 2022; Bianjiang et al., 2025). However, substantial heterogeneity exists across studies, reflecting variation in program structure, instructor expertise, participant motivation, and outcome measurement. Individual participant data meta-analyses further demonstrate marked interindividual variability, with some participants showing minimal or no empathic change (Galante et al., 2023).

Neuroimaging studies provide converging mechanistic evidence, showing mindfulness-related changes in anterior insula thickness, prefrontal connectivity, and neural markers of emotional regulation (Calderone et al., 2024; Bashir et al., 2025). Nevertheless, the durability of effects depends on continued practice, and attrition rates remain a practical limitation in real-world implementation (Conversano et al., 2020; Cooper et al., 2020).

### 6.2 Compassion-focused interventions

Compassion-focused therapy and related interventions explicitly train concern for others’ suffering and motivation to alleviate it. The most comprehensive recent meta-analysis of compassion-focused therapy with clinical populations found small to large effect sizes for improvements in self-compassion (0.19–0.90), self-criticism (0.15–0.72), and depression (0.24–0.25) (Millard et al., 2023; Kim et al., 2021). A 2025 systematic review found consistent improvements in self-compassion (*g* = 0.23–4.14) and reductions in self-criticism (*g* = 0.29–1.56) in clinical populations (Brown and Ashcroft, 2025; Martins et al., 2025).

### 6.3 Virtual reality–based perspective-taking

Virtual reality (VR) interventions aim to enhance empathy by immersing participants in simulated social experiences. Meta-analytic evidence demonstrates that VR produces moderate short-term improvements in emotional empathy (*d* = 0.51, 95% CI: 0.15–0.88), while effects on cognitive empathy are inconsistent (*d* = 0.21, 95% CI: −0.37–0.79) (Schutte and Stilinović, 2017; Martingano et al., 2021). A systematic review found that emotional empathy increases immediately after exposure but returns to baseline levels over time (Lee et al., 2024). VR shows promise in healthcare education and stigma reduction (Tay et al., 2025; Pira et al., 2025).

### 6.4 Educational and professional training programs

Social–emotional learning (SEL) programs represent the most robust real-world applications of empathy enhancement. A contemporary meta-analysis of 424 studies from 53 countries involving 575,361 students confirmed that SEL programs improve social and emotional skills, attitudes, behavior, and academic performance (Cipriano et al., 2023; Shi and Cheung, 2024). In healthcare education, meta-analyses indicate moderate improvements in empathy following structured training (d = 0.58), with interactive approaches outperforming didactic methods (Paulus and Meinken, 2022; Araújo et al., 2025) (Table 5). Large-scale healthcare and cross-cultural reviews further confirm that empathy training improves communication quality and patient experience across diverse sociocultural contexts (Sullivan-Detheridge et al., 2024; Yuan et al., 2025).

**Table 5:** Literature-derived translational readiness summaries for behavioral interventions.

Intervention type	Primary context	Reported effect size	Durability	Readiness	Key sources
Mindfulness-based training	Clinical; professional	*d* = 0.37 (95% CI: 0.16–0.58)	Practice-dependent	High	Hu et al. (2022) and Galante et al. (2023)
Compassion-focused therapy	Clinical populations	*g* = 0.23–4.14	Moderate with reinforcement	Moderate–High	Millard et al. (2023) and Brown and Ashcroft (2025)
VR perspective-taking	Education; healthcare	*d* = 0.51 (emotional empathy)	Low–moderate	Moderate	Martingano et al. (2021) and Lee et al. (2024)
SEL programs	Schools (K–12)	Small–moderate	High (curriculum-embedded)	High	Cipriano et al. (2023)
Healthcare empathy training	Medical education	*d* = 0.58	Moderate	High	Paulus and Meinken (2022) and Araújo et al. (2025)

## 7 Clinical applications and evidence-based outcomes

Clinical applications of empathy modulation technologies span multiple healthcare domains, with the strongest evidence base in healthcare provider training and educational settings. Recent systematic reviews reveal consistent benefits across diverse populations and intervention types, though implementation challenges and population-specific factors require careful consideration.

### 7.1 Healthcare provider training

A recent systematic review of 455 studies involving 470 analyses found that greater empathy is associated with better clinical outcomes and patient care experiences (Nembhard et al., 2023). A meta-analysis of 13 empathy training studies (*N* = 1,315) demonstrated an overall moderate effect size (*d* = 0.58) for empathy training effectiveness (Paulus and Meinken, 2022). A cluster randomized controlled trial in Ethiopian treatment centers showed sustained medium-to-large effects (*d* = 0.55 to 0.60) over 3 months, while simulation-based interventions showed effect sizes ranging from *d* = 0.46 (self-reports) to *d* = 1.27 (independent observers) (Chua et al., 2021; Hurissa et al., 2023).

Improved empathy among providers is associated with better diagnostic accuracy, increased medication adherence, lower malpractice risk, reduced patient anxiety and distress, and enhanced outcomes in chronic disease management. However, training effects frequently decline without reinforcement, and implementation challenges include institutional cultures that prioritize efficiency over patient interaction and systematic disparities in the quality of empathetic care for patients from lower socioeconomic backgrounds (Roberts et al., 2021; Byrne et al., 2024).

### 7.2 Educational and mental health applications

Educational systems provide a critical developmental window for cultivating empathy. Virtual patients through standardized scenarios ensure consistency and reproducibility while offering safe learning opportunities (Yamada et al., 2025). In mental health settings, empathy is foundational to the therapeutic alliance, one of the strongest predictors of treatment success. Higher therapist empathy predicts better client engagement, greater treatment adherence, and improved outcomes (Moudatsou et al., 2020). The relationship between trauma and empathy is complex, requiring tailored intervention approaches (Greenberg et al., 2018; Levy et al., 2019) (Table 6).

**Table 6:** Clinical translation readiness by intervention type.

Intervention	Research maturity	Clinical readiness	Implementation barriers	Recommended next steps
Healthcare empathy training	High	High	Institutional culture, sustainability	Implementation science research
SEL programs	High	High	Teacher training, cultural adaptation	Scale-up and sustainability research
Compassion-focused therapy	High	Moderate	Adherence, dose–response	Effectiveness trials
VR perspective-taking	Moderate	Moderate	Effect duration, targeting	Longitudinal follow-up
Neurostimulation (TMS/tDCS)	Moderate	Low	Individual variability, duration	Personalized targeting trials
LIFU/TIS	Low–Moderate	Very Low	Early development, safety data	Safety studies; mechanism trials
Pharmacological (oxytocin)	Moderate	Low	Small effects, genetic moderation	Genetically stratified trials

## 8 Ethical considerations in empathy modulation

The deliberate modulation of empathy raises distinct ethical concerns that extend beyond those associated with other cognitive or affective interventions. Because empathy is closely linked to emotional identity, moral judgment, and social behavior, interventions that alter empathic processing have the potential to affect autonomy, authenticity, and interpersonal relationships. UNESCO’s forthcoming 2025 Recommendation on the Ethics of Neurotechnology provides the first global framework addressing neurotechnologies that can “directly access, manipulate and emulate the structure of the brain” (UNESCO, 2025; Ramanathan, 2025). Recent international guidelines and ethical analyses emphasize that empathy-modulating interventions require careful governance, transparency, and respect for individual and cultural variability (Niles et al., 2023; Murphy et al., 2025).

### 8.1 Informed consent and durability of effects

A central ethical challenge concerns informed consent, particularly when interventions may produce enduring changes in emotional processing or value-based decision-making. Unlike transient cognitive enhancements, empathy-related changes may persist beyond the intervention period (Winter et al., 2020). Consent processes must include explicit discussion of uncertainty regarding duration, reversibility, and downstream social consequences. Dynamic consent models—in which participants are periodically re-informed and allowed to reassess participation—have been proposed as a safeguard (Young et al., 2022).

### 8.2 Vulnerability, coercion, and power asymmetries

Empathy modulation interventions are often deployed in contexts characterized by power asymmetries, including healthcare, education, military, and criminal justice settings. In such contexts, there is a risk that individuals may feel pressured to undergo interventions framed as beneficial or corrective (Sergiou et al., 2020; Beaudry et al., 2021; Roncero et al., 2025). Ethical implementation requires that participation be genuinely voluntary and that refusal carry no punitive consequences.

### 8.3 Neurodiversity and individual difference considerations

Contemporary research increasingly challenges deficit-based models of empathy, particularly in relation to autism spectrum conditions. The double empathy problem emphasizes that social misunderstandings arise bidirectionally between autistic and non-autistic individuals, rather than reflecting a unidirectional deficit (Cheang et al., 2025). Qualitative and quantitative studies demonstrate that autistic individuals often show intact or heightened empathy within neurodivergent peer groups (Watts et al., 2025). Ethical frameworks must respect neurodiversity and prioritize self-defined goals rather than externally imposed norms. Interventions should be co-designed with neurodivergent communities (Camilleri et al., 2025).

### 8.4 Cross-cultural contexts and misuse risks

Empathy is expressed and valued differently across cultures, shaped by social norms, religious beliefs, and historical context. Cross-cultural research demonstrates substantial variation in empathic expression and moral priorities (Eichbaum et al., 2023; Jami et al., 2024; Sullivan-Detheridge et al., 2024). There is also concern regarding potential misuse of empathy-modulating technologies for manipulation, persuasion, or social control. International governance frameworks emphasize preventing exploitative or coercive applications (UNESCO, 2025; Ramanathan, 2025) (Table 7).

**Table 7:** Population-specific ethical and clinical considerations.

Population	Promising interventions	Special considerations	Evidence quality
Healthcare providers	Mindfulness, simulation, communication skills	Time constraints, institutional culture, burnout prevention	High (multiple meta-analyses)
Autism spectrum	Individualized, neurodiversity-affirming approaches	Respect for neurodiversity, double empathy problem, community involvement	Moderate (paradigm shift ongoing)
Criminal justice	Perspective-taking, restorative justice	Voluntary participation essential, coercion concerns, motivation assessment	Low to moderate (limited RCTs)
Educational settings	SEL programs, VR, teacher training	Cultural adaptation, developmental appropriateness, teacher capacity	High (large global meta-analyses)
Mental health conditions	Trauma-informed, therapeutic alliance enhancement	Condition-specific adaptations, capacity assessment, emotional safety	Variable by condition
Children/adolescents	Age-appropriate SEL, developmental scaffolding	Evolving consent, developmental timing, parental involvement	High for SEL; limited for biological
Cross-cultural contexts	Culturally adapted interventions, local partnership	Avoid Western-centric frameworks, involve community leaders	Moderate (growing literature)

## 9 Conclusion and future directions

This hybrid narrative–scoping review synthesizes evidence across pharmacological, neurostimulation, and behavioral approaches to empathy modulation. The accumulated evidence indicates that empathy is biologically and psychologically modifiable, but that current interventions yield modest, heterogeneous, and often transient effects. Behavioral interventions—particularly mindfulness-based and compassion-focused programs—demonstrate the strongest combination of scalability, safety, and ecological validity, though effect sizes remain small to moderate (*d* = 0.37 for mindfulness) and depend on sustained engagement.

Pharmacological approaches provide valuable mechanistic insights but face substantial translational barriers. Intranasal oxytocin produces small, context-dependent effects (d = 0.24) that do not generalize reliably, while MDMA-assisted therapy demonstrates large effects (d ≈ 0.91) within tightly controlled psychotherapeutic settings but remains constrained by regulatory, ethical, and safety considerations following FDA rejection in August 2024. Neurostimulation techniques offer causal leverage over empathy-related circuits, particularly for cognitive empathy (*d* ≈ 0.18–0.20 for TMS), yet effects are short-lived and highly variable, limiting standalone clinical utility.

Future progress in empathy modulation will require several advances. First, larger and better-powered trials with standardized outcome measures are essential to establish reliable effect-size estimates—the field requires immediate prioritization of large-scale replication studies with at least 500 participants. Second, greater emphasis on ecological validity—including behavioral observation, longitudinal follow-up, and real-world outcomes—is needed to bridge the gap between laboratory findings and everyday social functioning. Third, integrative approaches combining behavioral training with biological or technological adjuncts may offer synergistic benefits, though such strategies must be evaluated cautiously.

**Figure 3:**
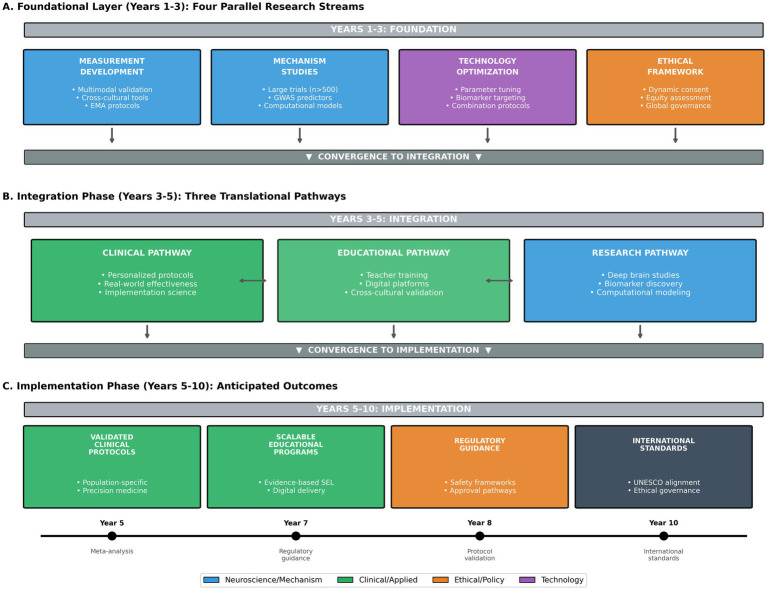
Research roadmap: priorities for translational empathy science. This figure presents a strategic roadmap for advancing empathy modulation research toward clinical translation, organized as a hierarchical flowchart with temporal milestones. **(A)** Shows the foundational layer (Years 1–3) comprising four parallel research streams: (1) Measurement development, including multimodal assessment validation, cross-cultural adaptation studies, and ecological momentary assessment protocols; (2) Mechanism studies, including large-scale replication trials (*n*500), genome-wide association studies for treatment response predictors, and computational modeling of empathy circuits; (3) Technology optimization, including parameter optimization for neurostimulation, biomarker-guided targeting development, and combination protocol testing; and (4) Ethical framework development, including dynamic consent model implementation, equity impact assessments, and international governance coordination. **(B)** Depicts the integration phase (Years 3–5) where these streams converge into three translational pathways: clinical pathway (personalized intervention protocols, real-world effectiveness trials, implementation science studies), educational pathway (teacher training program development, digital platform integration, cross-cultural validation), and research pathway (deep brain mechanism studies with emerging technologies, neural biomarker discovery, computational empathy modeling). **(C)** Shows the implementation phase (Years 5–10) with anticipated outcomes including validated clinical protocols for specific populations, scalable educational interventions with demonstrated effectiveness, regulatory guidance for empathy neurotechnologies, and international standards for ethical implementation. Arrows between elements indicate dependencies and feedback loops. Color coding distinguishes research domains: blue for neuroscience/mechanism studies, green for clinical/applied research, orange for ethical/policy development, and purple for technology development. This figure is a conceptual synthesis intended to guide research prioritization and is not derived from primary data.

Finally, ethical governance must remain central to the development and deployment of empathy-modulating interventions. Respect for autonomy, neurodiversity, cultural context, and voluntary participation is essential to ensure that efforts to enhance empathy contribute to individual well-being and social cohesion rather than coercion or misuse. With these foundations, empathy modulation may ultimately help reduce human suffering, enhance social cohesion, and improve outcomes across clinical, educational, and societal domains (Figure 3).

## References

[ref1] Abellaneda-Pérez K. Potash R. M. Pascual-Leone A. Sacchet M. D. (2024). Neuromodulation and meditation: a review and synthesis toward promoting well-being and understanding consciousness and brain. Neurosci. Biobehav. Rev. 166:105862. doi: 10.1016/j.neubiorev.2024.105862, 39186992

[ref2] Allen M. Frank D. Glen J. C. Fardo F. Callaghan M. F. Rees G. . (2017). Insula and somatosensory cortical myelination and iron markers underlie individual differences in empathy. Sci. Rep. 7:43316. doi: 10.1038/srep43316, 28256532 PMC5335674

[ref3] Araújo L. I. Ferreira E. S. Santos J. C. . (2025). The role of training and education for enhancing empathy among healthcare students: a systematic review of randomized controlled trials. BMC Med. Educ. 25:256. doi: 10.1186/s12909-025-06237-2 40170003 PMC11959860

[ref4] Badran B. W. Caulfield K. A. Stomberg-Firestein S. Summers P. M. Dowdle L. T. Savoca M. . (2020). Sonication of the anterior thalamus with MRI-guided transcranial focused ultrasound alters pain thresholds in healthy adults. Brain Stimul. 13, 1805–1812. doi: 10.1016/j.brs.2020.10.007 33127579 PMC7888561

[ref5] Bahji A. Forth E. Yang C. C. Khalifa N. (2021). Transcranial direct current stimulation for empathy: a systematic review and meta-analysis. Soc. Neurosci. 16, 232–255. doi: 10.1080/17470919.2021.1879203, 33567964

[ref6] Bakermans-Kranenburg M. J. van IJzendoorn M. H. (2013). Sniffing around oxytocin: review and meta-analyses of trials in healthy and clinical groups with implications for pharmacotherapy. Transl. Psychiatry 3:e258. doi: 10.1038/tp.2013.34, 23695233 PMC3669921

[ref7] Bashir K. Edstrom S. B. Barlow S. J. Gainer D. Lewis J. D. (2025). Loving-kindness meditation: systematic review of neuroimaging correlates in long-term practitioners and clinical implications. Brain Behav. 15:e70372. doi: 10.1002/brb3.70372, 40022190 PMC11870839

[ref8] Beaudry G. Yu R. Perry A. E. Fazel S. (2021). Effectiveness of psychological interventions in prison to reduce recidivism: a systematic review and meta-analysis of randomised controlled trials. Lancet Psychiatry 8, 759–773. doi: 10.1016/S2215-0366(21)00170-X, 34419185 PMC8376657

[ref9] Bianjiang Z. Jianchun Z. Xiaoyu S. Jian Y. (2025). Mind-body intervention for post-traumatic stress disorder in adolescents: a systematic review and network meta-analysis. BMC Psychiatry 25:178. doi: 10.1186/s12888-025-06543-1 40001042 PMC11863833

[ref10] Brooker B. H. Jeon J. Goldstein Ferber S. Bhatt R. S. Decety J. Bhatt M. A. . (2024). Neural correlates of empathy across species: evolutionary insights. Trends Cogn. Sci. 28, 312–325. doi: 10.1016/j.tics.2024.01.003

[ref11] Brown N. Ashcroft K. (2025). The effectiveness of compassion-focused therapy for the three flows of compassion, self-criticism, and shame in clinical populations: a systematic review. Behav. Sci. 15:1031. doi: 10.3390/bs15081031, 40867388 PMC12382812

[ref12] Byrne M. Campos C. Daly S. Lok B. Miles A. (2024). The current state of empathy, compassion and person-centred communication training in healthcare: an umbrella review. Patient Educ. Couns. 119:108063. doi: 10.1016/j.pec.2023.108063, 38008647

[ref13] Calderone A. Latella D. Impellizzeri F. de Pasquale P. Famà F. Quartarone A. . (2024). Neurobiological changes induced by mindfulness and meditation: a systematic review. Biomedicine 12:2613. doi: 10.3390/biomedicines12112613, 39595177 PMC11591838

[ref14] Camilleri L. J. Maras K. Brosnan M. (2025). Self-set goals: autistic adults facilitating their self-determination through digitally mediated social stories. Autism Adulthood 7, 25–38. doi: 10.1089/aut.2023.0122, 40151659 PMC11937795

[ref15] Chander R. J. Mather K. A. Cleary R. Grainger S. A. Kochan N. A. Henry J. D. . (2022). The influence of rs53576 polymorphism in the oxytocin receptor gene on empathy by subtype and ethnicity: a systematic review and meta-analysis. Rev. Neurosci. 33, 43–57. doi: 10.1515/revneuro-2020-0120 33892530

[ref16] Cheang R. T. Skjevling M. Blakemore A. I. McEwen F. S. Rijsdijk F. Happé F. . (2025). Do you feel me? Autism, empathic accuracy and the double empathy problem. Autism 29, 2315–2327. doi: 10.1177/13623613251316534 38757626 PMC12332230

[ref17] Chou Y. Chen T. Y. (2021). Disruption on right temporoparietal junction with transcranial magnetic stimulation affects moral judgment. Neuropsychologia 157:107858. doi: 10.1016/j.neuropsychologia.2021.107858 33857530

[ref18] Christensen M. C. Ren H. Fagiolini A. (2022). Emotional blunting in patients with depression. Part I: clinical characteristics. Ann. General Psychiatry 21:10. doi: 10.1186/s12991-022-00387-1, 35379283 PMC8981644

[ref19] Christov-Moore L. Simpson E. A. Coudé G. Grigaityte K. Iacoboni M. Ferrari P. F. (2014). Empathy: gender effects in brain and behavior. Neurosci. Biobehav. Rev. 46 Pt 4, 604–627. doi: 10.1016/j.neubiorev.2014.09.001, 25236781 PMC5110041

[ref20] Chua J. Y. X. Ang E. Lau S. T. L. Shorey S. (2021). Effectiveness of simulation-based interventions at improving empathy among healthcare students. Nurse Educ. Today 104:105000. doi: 10.1016/j.nedt.2021.105000, 34146845

[ref21] Cipriano C. Strambler M. J. Naples L. H. Ha C. Kirk M. Durlak J. . (2023). The state of evidence for social and emotional learning: a contemporary meta-analysis. Child Dev. 94, 1181–1204. doi: 10.1111/cdev.13968 37448158

[ref22] Conversano C. Ciacchini R. Orrù G. Di Giuseppe M. Gemignani A. Poli A.. (2020). Mindfulness, compassion, and self-compassion among health care professionals. Front. Psychol. 11:1683. doi: 10.3389/fpsyg.2020.01683 32849021 PMC7412718

[ref23] Cooper D. Yap K. O'Brien M. Scott I. (2020). Mindfulness and empathy among counseling and psychotherapy professionals. Mindfulness 11, 2243–2257. doi: 10.1007/s12671-020-01446-6

[ref24] Cox S. S. Kearns A. M. Woods S. K. Brown B. J. Brown S. J. Reichel C. M. (2022). The role of the anterior insula during targeted helping behavior in male rats. Sci. Rep. 12:3315. doi: 10.1038/s41598-022-07365-3, 35228625 PMC8885669

[ref25] Craig A. D. (2009). How do you feel—now? The anterior insula and human awareness. Nat. Rev. Neurosci. 10, 59–70. doi: 10.1038/nrn2555, 19096369

[ref26] Decety J. Bartal I. B.-A. Uzefovsky F. Knafo-Noam A. (2016). Empathy as a driver of prosocial behaviour: highly conserved neurobehavioural mechanisms across species. Philos. Trans. R. Soc. Lond. Ser. B Biol. Sci. 371:20150077. doi: 10.1098/rstb.2015.0077, 26644596 PMC4685523

[ref27] Eichbaum Q. Barbeau-Meunier C. A. White M. Côté L. Hausse L. de Lalouvière G. . (2023). Empathy across cultures—one size does not fit all. Adv. Health Sci. Educ. 28, 643–657. doi: 10.1007/s10459-022-10172-6 PMC9491267 36129550

[ref28] Ferguson H. J. De Lillo M. Woodrow-Hill C. Foley R. Bradford E. E. F. (2024). Neural empathy mechanisms are shared for physical and social pain, and increase from adolescence to older adulthood. Soc. Cogn. Affect. Neurosci. 19:nsae080. doi: 10.1093/scan/nsae080, 39492751 PMC11630255

[ref29] Galante J. Friedrich C. Aeamla-Or N. Arts I. Bögels S. M. Buitelaar J. K. . (2023). Systematic review and individual participant data meta-analysis of randomized controlled trials assessing mindfulness-based programs for mental health promotion. Nat. Ment. Health 1, 462–476. doi: 10.1038/s44220-023-00081-1 37867573 PMC7615230

[ref30] Gazzola V. (2018). HelpUS: pioneering focused ultrasounds as a new non-invasive deep brain stimulation for causal investigation of empathy. European Research Council (ERC) Advanced Grant No. 758703. European Research Council, Brussels, Belgium.

[ref31] Greenberg D. M. Baron-Cohen S. Rosenberg N. Fonagy P. Rentfrow P. J. (2018). Elevated empathy in adults following childhood trauma. PLoS One 13:e0203886. doi: 10.1371/journal.pone.0203886, 30281628 PMC6169872

[ref32] Haynes B. Ortega H. K. Damisah E. C. Bhatt M. A. Bickel S. Mehta A. D. . (2025). The neural basis of emotional generalization in empathy. [Epubh ahead of print] doi: 10.1101/2025.10.18.683207

[ref33] Holleman G. A. Hooge I. T. C. Kemner C. Hessels R. S. (2020). The 'real-world approach' and its problems: a critique of the term ecological validity. Front. Psychol. 11:721. doi: 10.3389/fpsyg.2020.00721, 32425850 PMC7204431

[ref34] Hu Z. Wen Y. Wang Y. Lin Y. Shi J. Yu Z. . (2022). Effectiveness of mindfulness-based interventions on empathy: a meta-analysis. Front. Psychol. 13:992575. doi: 10.3389/fpsyg.2022.992575, 36337535 PMC9632989

[ref35] Hurissa B. F. Koricha Z. B. Dadi L. S. (2023). Effect of empathy training on the empathy level of healthcare providers in Ethiopia. Front. Psychol. 14:1091605. doi: 10.3389/fpsyg.2023.1091605, 37284470 PMC10239930

[ref36] Jami P. Y. Walker D. I. Mansouri B. (2024). Interaction of empathy and culture: a review. Curr. Psychol. 43, 2965–2980. doi: 10.1007/s12144-022-03028-0

[ref37] Jiang Q. Zhuo L. Wang Q. Lin W. (2022). The neural basis of moral judgement for self and for others. Front. Hum. Neurosci. 16:919499. doi: 10.3389/fnhum.2022.919499, 35693541 PMC9184798

[ref38] Jungwirth J. von Rotz R. Dziobek I. Vollenweider F. X. Preller K. H. (2025). Psilocybin increases emotional empathy in patients with major depression. Mol. Psychiatry 30, 2665–2672. doi: 10.1038/s41380-024-02875-0, 39695323 PMC12092279

[ref39] Kim S. A. Hamann S. Kim S. H. (2021). Neurocognitive mechanisms underlying improvement of prosocial responses by a novel implicit compassion promotion task. NeuroImage 240:118333. doi: 10.1016/j.neuroimage.2021.118333, 34229063

[ref40] Kim M. G. Yu K. Yeh C. Y. Cannesson M. Bhatt D. L. He B. . (2024). Low-intensity transcranial focused ultrasound suppresses pain by modulating pain-processing brain circuits. Blood 144, 1101–1115. doi: 10.1182/blood.2024024180 38976875 PMC11406192

[ref41] Kogler L. Müller V. I. Chang A. Eickhoff S. B. Fox P. T. Gur R. C. . (2020). Do i feel or do i know? Neuroimaging meta-analyses on the multiple facets of empathy. Cortex 129, 268–285. doi: 10.1016/j.cortex.2020.04.031 PMC7390692 32562973

[ref42] Kuroda K. O. Kato M. Tsuneoka Y. Shoji H. Nishimori K. Miyata T. . (2024). Evolutionary neurobiology of parenting and empathy. Neurosci. Biobehav. Rev. 158:105543. doi: 10.1016/j.neubiorev.2024.105543 38220036

[ref43] Langley C. Armand S. Luo Q. Savulich G. Segerberg T. Søndergaard A. . (2023). Chronic escitalopram in healthy volunteers has specific effects on reinforcement sensitivity. Neuropsychopharmacology 48, 664–670. doi: 10.1038/s41386-022-01523-x 36683090 PMC9938113

[ref44] Lee Y. Shin H. Gil Y. H. (2024). Measurement of empathy in virtual reality with head-mounted displays: a systematic review. IEEE Trans. Vis. Comput. Graph. 30, 2485–2495. doi: 10.1109/TVCG.2024.3372074, 38437085

[ref45] Legon W. Strohman A. In A. Payne B. (2024). Noninvasive neuromodulation of subregions of the human insula differentially affect pain processing and heart-rate variability. Pain 165, 1625–1641. doi: 10.1097/j.pain.0000000000003177, 38314779 PMC11189760

[ref46] Levy J. Goldstein A. Feldman R. (2019). The neural development of empathy is sensitive to caregiving and early trauma. Nat. Commun. 10:1905. doi: 10.1038/s41467-019-09927-y, 31015471 PMC6478745

[ref47] Liang X. Wang Y. Chen H. Zhang L. Liu M. Wu J. . (2025). Dopaminergic pathways and developmental trajectories of empathy. Dev. Cogn. Neurosci. 71:101456. doi: 10.1016/j.dcn.2025.101456

[ref48] Lima F. F. Osório F. L. (2021). Empathy: assessment instruments and psychometric quality. Front. Psychol. 12:781346. doi: 10.3389/fpsyg.2021.781346, 34899531 PMC8653810

[ref49] Lu Y. Lau S. K. (2025). The ecological validity of laboratory experiments in soundscape and landscape research: a systematic review and meta-analysis. Appl. Acoust. 232:110582. doi: 10.1016/j.apacoust.2024.110582

[ref50] Ma H. Cai M. Wang H. (2021). Emotional blunting in patients with major depressive disorder. Front. Psych. 12:792960. doi: 10.3389/fpsyt.2021.792960, 34970173 PMC8712545

[ref51] Marsh N. Marsh A. A. Lee M. R. Hurlemann R. (2021). Oxytocin and the neurobiology of prosocial behavior. Neuroscientist 27, 604–619. doi: 10.1177/1073858420960111, 32981445 PMC8640275

[ref52] Martin E. Aubry J. F. Bhatt S. Bhatt D. L. Bystritsky A. Cohen S. L. . (2024). Updated international consensus on safety for transcranial ultrasonic neuromodulation. Brain Stimul. 17, 674–691. doi: 10.1016/j.brs.2024.05.006

[ref53] Martingano A. J. Hererra F. Konrath S. (2021). Virtual reality improves emotional but not cognitive empathy: a meta-analysis. Technol. Mind Behav. 2:TMB0000034. doi: 10.1037/tmb0000034

[ref54] Martins D. Gomes A. R. Lopes S. Pinto E. Ferreira A. Cunha M. . (2025). Workplace-based compassion interventions: a systematic review. J. Occup. Health Psychol. 30, 45–62. doi: 10.1037/ocp0000345

[ref55] Matsushita H. Nishiki T. (2025). Human social behavior and oxytocin: molecular and neuronal mechanisms. Neuroscience 570, 48–54. doi: 10.1016/j.neuroscience.2025.01.015, 39961388

[ref56] Millard L. A. Wan M. W. Smith D. M. Wittkowski A. (2023). The effectiveness of compassion focused therapy with clinical populations: a systematic review and meta-analysis. J. Affect. Disord. 326, 168–192. doi: 10.1016/j.jad.2023.01.010, 36649790

[ref57] Miller J. G. Xia G. Hastings P. D. (2020). Right temporoparietal junction involvement in autonomic responses to the suffering of others. Front. Hum. Neurosci. 14:7. doi: 10.3389/fnhum.2020.00007, 32047426 PMC6997337

[ref58] Mitchell J. M. Bogenschutz M. Lilienstein A. Harrison C. Kleiman S. Parker-Guilbert K. . (2021). MDMA-assisted therapy for severe PTSD: a randomized, double-blind, placebo-controlled phase 3 study. Nat. Med. 27, 1025–1033. doi: 10.1038/s41591-021-01336-3, 33972795 PMC8205851

[ref59] Mitchell J. M. Ot'alora G. M. van der Kolk B. Shannon S. Bogenschutz M. Gelfand Y. . (2023). MDMA-assisted therapy for moderate to severe PTSD: a randomized, placebo-controlled phase 3 trial. Nat. Med. 29, 2473–2480. doi: 10.1038/s41591-023-02565-4, 37709999 PMC10579091

[ref60] Modak A. Fitzgerald P. B. Engel S. Rogasch N. C. Segrave R. A. Hoy K. E. . (2024). Temporal interference stimulation enhances episodic memory. J. Cogn. Enhanc. 8, 112–125. doi: 10.1007/s41465-024-00298-1

[ref61] Moudatsou M. Stavropoulou A. Philalithis A. Koukouli S. (2020). The role of empathy in health and social care professionals. Healthcare 8:26. doi: 10.3390/healthcare8010026, 32019104 PMC7151200

[ref62] Murphy K. Bhatt S. Martin E. Aubry J. F. Bhatt D. L. Bystritsky A. . (2025). Safety guidelines for transcranial ultrasonic stimulation: updated ITRUSST recommendations. Brain Stimul. 18, 45–58. doi: 10.1016/j.brs.2025.01.002

[ref63] Nembhard I. M. David G. Ezzeddine I. Bhatt D. L. Bhatt S. Bodenheimer T. . (2023). A systematic review of research on empathy in health care. Health Serv. Res. 58, 250–263. doi: 10.1111/1475-6773.14102 35765156 PMC10012244

[ref64] Niles B. Lang A. Olff M. (2023). Complementary and integrative interventions for PTSD. Eur. J. Psychotraumatol. 14:2247888. doi: 10.1080/20008066.2023.2247888, 37655624 PMC10478588

[ref65] Paradiso E. Gazzola V. Keysers C. (2021). Neural mechanisms necessary for empathy-related phenomena across species. Curr. Opin. Neurobiol. 68, 107–115. doi: 10.1016/j.conb.2021.02.005, 33756399

[ref66] Paulus C. M. Meinken S. (2022). The effectiveness of empathy training in health care: a meta-analysis. Int. J. Med. Educ. 13, 1–9. doi: 10.5116/ijme.619e.c2b3, 35092671 PMC8995011

[ref67] Pellow C. O’Reilly M. A. Bhatt S. Bhatt D. L. Hynynen K. Bhatt M. A. . (2024). Mechanistic insights into focused ultrasound neuromodulation. Nat. Rev. Neurosci. 25, 412–428. doi: 10.1038/s41583-024-00812-2

[ref68] Pira G. L. Ruini C. Vescovelli F. Gatta M. Porro G. Vanini G. . (2025). Could empathy be taught? The role of advanced technologies to foster empathy in medical students. J. Med. Syst. 49:6. doi: 10.1007/s10916-024-02134-5 39806022 PMC11729101

[ref69] Ramanathan M. (2025). The UNESCO draft recommendations on ethics of neurotechnology—a commentary. Indian J Med Ethics 10, 89–91. doi: 10.20529/IJME.2025.012 40561423

[ref70] Reardon S. (2024). MDMA therapy for PTSD rejected by FDA panel. Nature. 629, 20–21. doi: 10.1038/d41586-024-01622-3, 38844808

[ref71] Rein B. Raymond K. Boustani C. Tuy S. Zhang J. St Laurent R. . (2024). MDMA enhances empathy-like behaviors in mice via 5-HT release in the nucleus accumbens. Sci. Adv. 10:eadl6554. doi: 10.1126/sciadv.adl6554, 38657057 PMC11042730

[ref72] Rinaldi C. Attanasio M. Valenti M. Mazza M. Keller R. (2021). Autism spectrum disorder and personality disorders: comorbidity and differential diagnosis. World J. Psychiatry 11, 1366–1386. doi: 10.5498/wjp.v11.i12.1366, 35070783 PMC8717043

[ref73] Roberts B. W. Puri N. K. Trzeciak C. J. Mazzarelli A. J. Mooney E. R. Trzeciak S.. (2021). Socioeconomic, racial and ethnic differences in patient experience of clinician empathy. PLoS One 16:e0247259. doi: 10.1371/journal.pone.0247259 33657153 PMC7928470

[ref74] Rolls E. T. (2019). The cingulate cortex and limbic systems for emotion, action, and memory. Brain Struct. Funct. 224, 3001–3018. doi: 10.1007/s00429-019-01945-2, 31451898 PMC6875144

[ref75] Roncero D. Moreno-Fernández R. D. Fernández-Moreno Á. (2025). Effectiveness of virtual reality interventions for aggression, anger and impulsiveness: a multilevel meta-analysis. Aggress. Violent Behav. 81:102034. doi: 10.1016/j.avb.2024.102034

[ref76] Rossi S. Antal A. Bestmann S. Bikson M. Brewer C. Brockmöller J. . (2021). Safety and recommendations for TMS use in healthy subjects and patient populations. Clin. Neurophysiol. 132, 269–306. doi: 10.1016/j.clinph.2020.10.003 33243615 PMC9094636

[ref77] Rütgen M. Pletti C. Tik M. Kraus C. Pfabigan D. M. Sladky R. . (2019). Antidepressant treatment, not depression, leads to reductions in behavioral and neural responses to pain empathy. Transl. Psychiatry 9:164. doi: 10.1038/s41398-019-0496-4, 31175273 PMC6555809

[ref78] Saccenti D. Lauro L. J. R. Crespi S. A. Vergallito A. Zanella M. Bolognini N. . (2024). Boosting psychotherapy with noninvasive brain stimulation. Neural Plast. 2024:7853199. doi: 10.1155/2024/7853199 39723244 PMC11669434

[ref79] Saxe R. Kanwisher N. (2003). People thinking about thinking people: the role of the temporo-parietal junction in 'theory of mind'. NeuroImage 19, 1835–1842. doi: 10.1016/S1053-8119(03)00230-1, 12948738

[ref80] Schutte N. S. Stilinović E. J. (2017). Facilitating empathy through virtual reality. Motiv. Emot. 41, 708–712. doi: 10.1007/s11031-017-9641-7

[ref81] Schwertfeger J. L. Beyer C. Hung P. Ahrens A. P. Leahy A. B. Cook N. E. . (2023). A map of evidence using tDCS to improve cognition in adults with TBI. Front. Neuroergonomics 4:1170473. doi: 10.3389/fnrgo.2023.1170473 PMC10790940 38234478

[ref82] Sergiou C. S. Woods A. J. Franken I. H. A. van Dongen J. D. M. (2020). Transcranial direct current stimulation to improve empathic abilities in forensic offenders: study protocol. Trials 21:263. doi: 10.1186/s13063-020-4166-1 32169111 PMC7069186

[ref83] Shamay-Tsoory S. G. Abu-Akel A. (2016). The social salience hypothesis of oxytocin. Biol. Psychiatry 79, 194–202. doi: 10.1016/j.biopsych.2015.07.020 26321019

[ref84] Shi J. Cheung A. C. K. (2024). Effective components of social emotional learning programs: a meta-analysis. J. Youth Adolesc. 53, 755–771. doi: 10.1007/s10964-023-01913-6, 38280178

[ref85] Singer T. Klimecki O. M. (2014). Empathy and compassion. Curr. Biol. 24, R875–R878. doi: 10.1016/j.cub.2014.06.054, 25247366

[ref86] Singer T. Seymour B. O'Doherty J. Kaube H. Dolan R. J. Frith C. D. (2004). Empathy for pain involves the affective but not sensory components of pain. Science 303, 1157–1162. doi: 10.1126/science.1093535, 14976305

[ref87] Son J. J. Erker T. D. Ward T. W. Datta A. Edwards D. J. Bikson M. . (2025). The polarity of high-definition transcranial direct current stimulation affects movement sequences. NeuroImage 306:121018. doi: 10.1016/j.neuroimage.2024.121018 39800171 PMC11829609

[ref88] Stark N. Bobadilla L. Michael P. Beeney J. E. Lane S. P. Wilson S.. (2023). A meta-analytic review of the relationship between empathy and oxytocin. Aggress. Violent Behav. 70:101828. doi: 10.1016/j.avb.2023.101828

[ref89] Sullivan-Detheridge J. H. Reifsnider E. Mengsteab M. Merie K. Staller J. Allen A. M. (2024). Cross cultural empathetic behavior in health care providers: a review of 3 countries. J. Prim. Care Community Health 15:21501319241226765. doi: 10.1177/21501319241226765, 38254300 PMC10807346

[ref90] Tay J. L. Qu Y. Lim L. Sim K. Subramaniam M. Chua H. C. . (2025). Impact of a virtual reality intervention on stigma, empathy, and attitudes toward patients with psychotic disorders. JMIR Ment. Health 12:e66925. doi: 10.2196/66925 39836956 PMC11795159

[ref91] Tian X. Zheng Z. Li R. Luo Y. J. Feng C. (2025). Neural signatures underlying the effect of social structure on empathy and altruistic behaviors. NeuroImage 315:121267. doi: 10.1016/j.neuroimage.2025.121267, 40368058

[ref92] UNESCO (2025). Draft text of the recommendation on the ethics of Neurotechnology (SHS/IGM-NEURO/2025/MAY/3): UNESCO.

[ref93] Vaslavski A. Gross A. H. Israel S. Peled-Avron L. (2025). The effect of MDMA administration on oxytocin concentration levels: a systematic review and meta-analysis. Neurosci. Biobehav. Rev. 177:106324. doi: 10.1016/j.neubiorev.2025.106324 40812728

[ref94] Vassiliadis P. Stiennon E. Windel F. Missey F. Gracia P. Hummel F. C. . (2024). Safety, tolerability and blinding efficiency of non-invasive deep transcranial temporal interference stimulation. J. Neural Eng. 21:024001. doi: 10.1088/1741-2552/ad2d32 38408385

[ref95] Violante I. R. Alania K. Cassarà A. M. Neufeld E. Acerbo E. Carron R. . (2023). Noninvasive temporal interference electrical stimulation of the human hippocampus. Nat. Neurosci. 26, 1994–2004. doi: 10.1038/s41593-023-01456-8, 37857775 PMC10620081

[ref96] Wang S. Liu N. Chen J. Zhang X. Li Y. Zhao H. . (2025). Efficacy and safety of transcranial temporal interference stimulation for improving negative symptoms in schizophrenia: a pilot study. Schizophr. Bull. doi: 10.1093/schbul/sbaf038

[ref97] Watts G. Crompton C. Grainger C. Milton D. Fletcher-Watson S. Sasson N. J. . (2025). A certain magic': autistic adults' experiences of interacting with other autistic people. Autism 29, 2239–2253. doi: 10.1177/13623613251312890 38829019 PMC12332227

[ref98] Wilson J. Trumbo M. Tesche C. (2021). Transcranial direct current stimulation improves empathy and emotion recognition in adults with autism spectrum disorder. NeuroRegulation 8, 87–95. doi: 10.15540/nr.8.2.87

[ref99] Winter R. Issa E. Roberts N. Norman R. I. Howick J.. (2020). Assessing the effect of empathy-enhancing interventions in health education: a systematic review. BMJ Open 10:e036471. doi: 10.1136/bmjopen-2019-036471 PMC7520826 32978187

[ref100] Wolfgang A. S. Fonzo G. A. Gray J. C. Mithoefer M. C. Yazar-Klosinski B. Emerson A. . (2025). MDMA and MDMA-assisted therapy. Am. J. Psychiatry 182, 79–103. doi: 10.1176/appi.ajp.20240583 39741438

[ref101] Yamada R. Futakawa K. Xu K. Kondo S. (2025). Using virtual patients to enhance empathy in medical students: a scoping review protocol. Syst. Rev. 14:52. doi: 10.1186/s13643-025-02469-1 40025554 PMC11871709

[ref102] Yang C. C. Khalifa N. Völlm B. (2018). The effects of repetitive transcranial magnetic stimulation on empathy: a systematic review and meta-analysis. Psychol. Med. 48, 737–750. doi: 10.1017/S0033291717002161, 28826416

[ref103] Young M. J. Bodien Y. G. Edlow B. L. (2022). Ethical considerations in clinical trials for disorders of consciousness. Brain Sci. 12:211. doi: 10.3390/brainsci12020211, 35203974 PMC8870384

[ref104] Yuan Y. Gao Z. Xiao W. (2025). The role of oxytocin in parental care. Endocrinology 166:bqaf129. doi: 10.1210/endocr/bqaf129, 40856229

[ref105] Zhang H. Xiong G. Cai S. Wu S. (2024). A causal role of right temporoparietal junction in prosocial learning: a transcranial direct current stimulation study. Neuroscience 538, 59–67. doi: 10.1016/j.neuroscience.2023.12.007, 38145822

